# Forces on the Incisor Teeth During Odontoplasty of the Cheek Teeth in Sedated Horses

**DOI:** 10.1177/08987564251336397

**Published:** 2025-05-05

**Authors:** Martin Ostmeier, Frank Schellenberger, Antonia Troillet, Doreen Scharner

**Affiliations:** 1 Department for horses, Faculty of veterinary medicine, University of Leipzig, Leipzig, Germany; 2 Tierarztpraxis Dr Frank Schellenberger, Waldkirch, Germany

**Keywords:** horse, mouth speculum, masticatory forces, incisors, dental treatment, odontoplasty

## Abstract

Mouth specula with incisor bite plates shift the pressure from all teeth exclusively to the incisors in the opening phase which leads to increased forces on dental, osseous, and soft tissue structures of the horse's head. The potentially pathological character of these forces is described clinically by single reports of maxilla and mandible fractures occurring during the use of opened mouth specula. This study describes these forces on incisor teeth in horses during odontoplasty of cheek teeth under sedation. Measurements were documented using a modified “Günther” mouth speculum fitted with a force transducer along the force axis. Forces were recorded for different incisor separation distances and for dental rasping at a defined incisor separation distance. The results showed a significant difference in the median loads on the incisors at 82 mm (198.88 N), 92 mm (214.18 N), and 102 mm (293.95 N) incisor separation distance, and between active treatment with a mechanical bur (173.28 N) and no active mechanical bur (237.81 N) with maximum peaks up to 3783.60 N. Increasing bodyweight and mandible length showed a correlation with increasing forces, whereas age, gender and regularity of dental treatments did not suggest any influence on force development.

## Introduction

In the recent history of equine dentistry an increase in routine oral examinations has been seen based on improved understanding of dental diseases and insight into the importance of preventative treatments.^1-3^

Most oral specula utilize a lever function with loading of incisor plates for safe oral examination as originally described in 1859.^4^ This mouth speculum design shifts the entire force necessary for opening the mouth on the incisor bite plates. Opening forces must overcome the contraction impulse of the masticatory muscle system. The consequences for the dental tissues, the periodontium and alveoli have not yet been examined and can only be assumed.

In the course of further development of equine dentistry, various mouth specula with different opening mechanisms and materials have been developed to ensure a better and safer dental treatment.^5^ Medical progress is enabling new treatment options, which can result in longer treatment times and strain in the involved structures.^6^ Single field reports describe potential incidences such as fractures of the mouth specula or fractures of the maxilla or mandible during dental treatments, which raise questions concerning the resulting forces on material and anatomical structures involved in the masticatory process.^7^

Up to now, studies have mainly focused on forces occurring in the region of the cheek teeth.^8-11^ Two studies also describe forces on the incisors but do not reflect the conditions during dental treatment.^8,9^ The aim of this study is to determine the forces on incisor teeth in horses during odontoplasty of the cheek teeth. Corrective procedures included rasping of pathological changes in the occlusal surface due to rostrocaudal malposition, such as pathological transverse ridges, protuberant teeth, diastemata, etc, in order to restore a physiological and functional cheek teeth row and remove sharp enamel points that have led to mucosal lesions. In severe cases of protuberant teeth, dental structure was removed until the staining of secondary dentine disappeared. Further, the influence of defined incisor separation distances (ISD) of the horse's jaw as well as age, gender, breed, body weight, mandible length and frequency of dental treatments on applied incisor teeth forces were analyzed.

It is hypothesized that the forces on the incisors are positively related to the ISD of the horse's jaw and that the stresses occurring throughout a dental treatment are higher while actively rasping the cheek teeth using a mechanical bur than during examination.

## Material and Methods

The study consisted of a field population of adult horses of various breeds from southern Germany presented for routine dental examinations in September and October 2021. Age, gender, breed, bodyweight, mandible length, and regularity of dental treatments were documented. Exclusion criteria were the necessity for tooth extraction, horses suffering from equine odontoclastic tooth resorption and hypercementosis with avoidance reactions upon palpation, and absence of one or more incisors. The study was approved by the Ethical Committee of the University of Leipzig (# 2/23).

Prior to positioning of the mouth speculum, all horses underwent a physical examination. Mandible length defined as distance between temporomandibular joint and interdental space of the mandibular first incisor teeth (301/401), weight by formula^12^ and regularity of dental treatments were recorded. The treatment was classified as recurrent if it was repeated at least once within 2 years.

**Figure 1.**
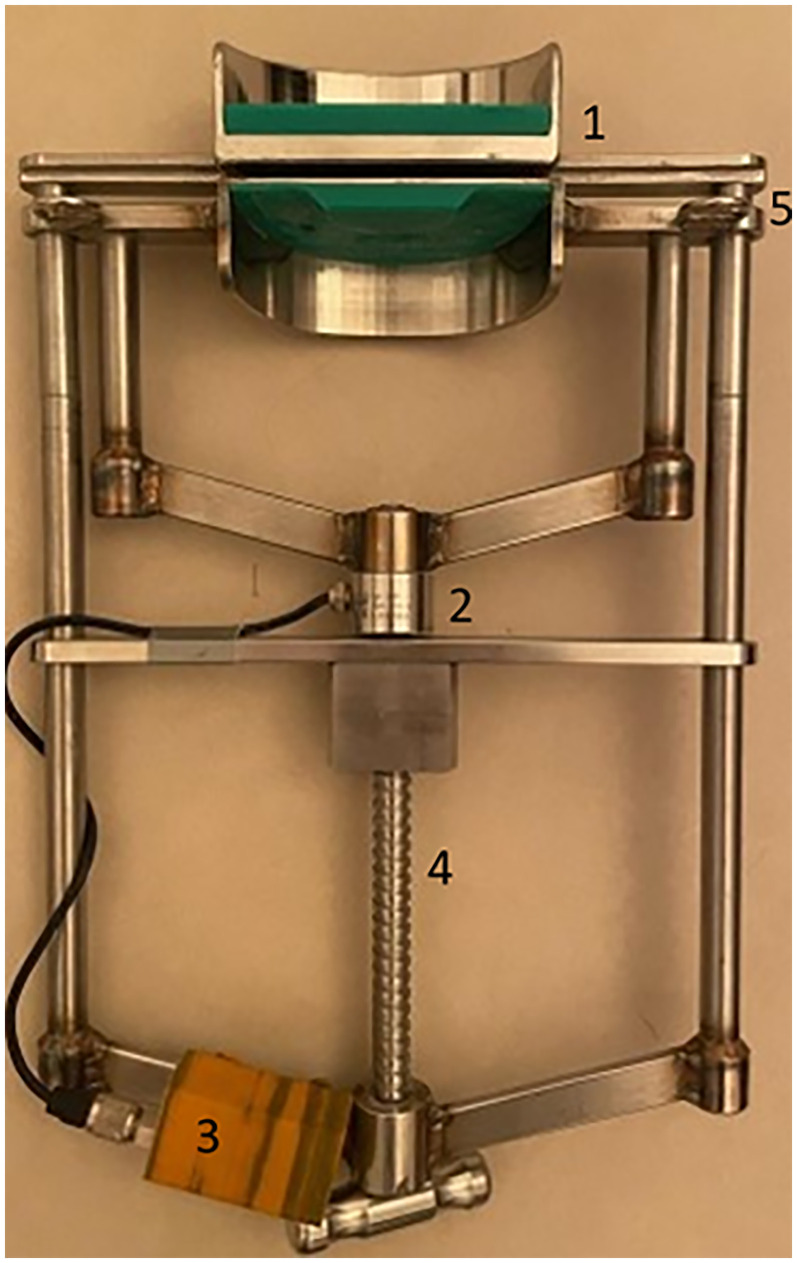
Modified “Günther” mouth speculum showing the angulated bite plates (1), the force transducer (2), the WLAN transmitter (3), the “screw device” (4) and the eyelets to fix the neck strap (5).

**Figure 2.**
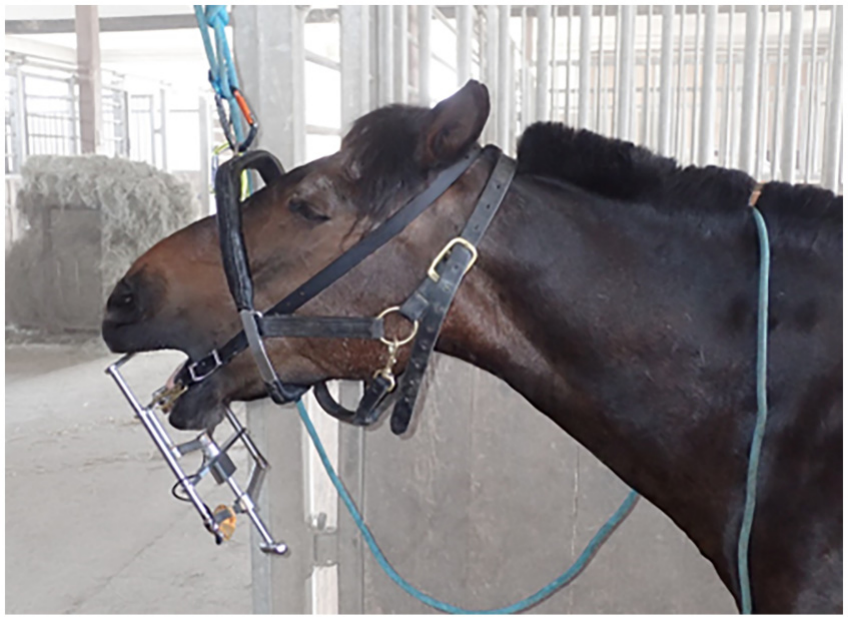
Positioning of the head within the halter during measurements and treatments. The modified “Günther” mouth speculum is held in position by the neck strap.

A modified “Günther” mouth speculum^a^ with exchangeable bite plates was used for the measurements (Figure 1). In all cases the lower and upper incisor bite plates were made of polyoxymethylene (POM). The upper incisor bite plate had an angulation of 4° and the lower incisor bite plate had an angulation of 16°. Positive angles are equivalent to a counterclockwise rotation of the bite plate plane from a horizontal line. The mandibular bite plate also had an antislip feature in the form of a raised central ledge. In addition, two eyelets were attached to the outside of the cross struts of the mandibular bite plate to hold the mouth speculum in the horse's mouth with the aid of a neck strap (Figure 2).

A force transducer type U9B^b^ with a maximum load of 10kN was installed in the mouth speculum between the mandibular bite plate and the screw device. Data derived from the tensile load cell were transmitted in kg to a receiver device every 8 milliseconds using a WLAN transmitter in a three-dimensional printed sheath. A software program written to visualize the measured data on a force/time axis allowed immediate classification of the predefined actions. A basic calibration of the force transducer was performed prior to start and after the end of the measurement series to verify and ensure the accuracy of the measured data. Due to an interaction of the force transducer with the external temperature, initial values were determined before each measurement without applying pressure to the bite plates while holding the mouth speculum vertically to avoid false results.

Different ISD of the mouth speculum were selected as fixed points for comparison based on a Hausmann mouth speculum^c^ at ratchet notches 2 and 3 out of 6, which correspond to an ISD of 82 mm (ratch 2) and 102 mm (ratch 3). A third measurement at an ISD of 92 mm was added for comparison.

An initial measurement was taken at the different defined distances for 5-10 s to determine the base force on the incisors with as little chewing as possible. Subsequently, the load at an ISD of 92 mm was documented during ongoing dental treatment, distinguishing between the period of an active dental grinding bur and none in the patient's oral cavity.

**Figure 3.**
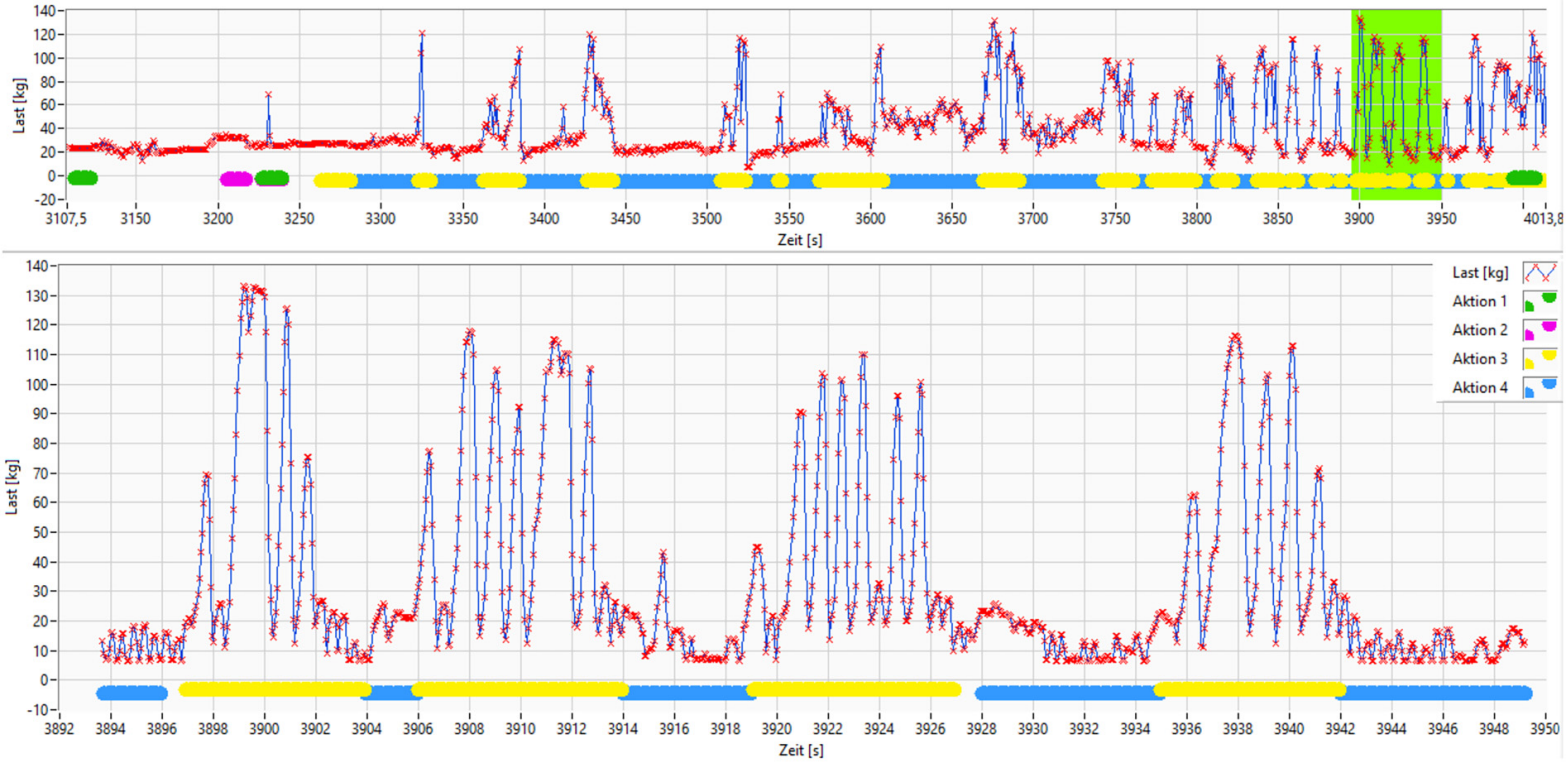
Original image of measurement program showing forces on incisors during dental treatment with an incisor separation distance of 92 mm. Values are demonstrated in a time (s)/force (kg) diagram. The upper diagram demonstrates the full time of treatment. The green boxed highlighted section is shown in the lower diagram in greater detail, with the blue underlined sections indicating the active mechanical bur and the yellow underlined sections indicating no active mechanical bur.

A halter for dental treatments was used to suspend the head at a consistent height relative to body size of each horse (Figure 2) and the measurements were performed in a sitting position by the veterinarian as described above. All force measurements were performed by one author (MO) before and during odontoplasty. Treatments took place at the home stables by the same specialist in equine dentistry (FS). All horses were sedated with detomidine and butorphanol based on their body weight and patients’ compliance to ensure safe examination and treatment, according to the attending veterinarian. Then the modified “Günther” mouth speculum with force transducer was fitted into position. The head was suspended using the special halter. Data representing forces on the incisors were documented and simultaneously manually assigned to the dental treatment actions being performed (Figure 3).

## Statistical Analysis

The statistical analysis of the data was carried out using IBM SPSS Statistics^d^. Descriptive statistics were calculated and boxplots were created. The data was analyzed for normal distribution using the Shapiro-Wilk test. For non-normally distributed data, the median and the interquartile range (IQR) was given. The Wilcoxon signed-rank test was used to compare paired samples of non-normally distributed data. For group comparisons of non-normally distributed, unrelated data, the Mann–Whitney *U* test was used. For group comparisons of non-normally distributed, linked data, the Friedman test was used. Correlations were calculated using the Spearman correlation coefficient for non-normally distributed data and are to be interpreted as follows: 0.8 ≤ ρ ≤ 1.0 very strong, 0.6 ≤ ρ < 0.8 strong, 0.4 ≤ ρ < 0.6 moderate, 0.2 ≤ ρ < 0.4 weak and 0.0 ≤ ρ < 0.2 very weak; (ρ represents the magnitude of the correlation coefficient). The significance level was set at *p *= .05 for all tests.

## Results

Measurements were performed on 78 horses (3 stallions, 34 geldings, 41 mares) including Warmbloods, Icelandic horses, Quarter horses, Thoroughbreds, Ponies and Draft horses, aged between 3 and 31 years with a median weight of 513.5 kg (IQR 1535 kg) (Table 1).

**Table 1.** Age and Weights of Each Cohort of Horses.

Breed	Number	Median age (Range) Y	Median weight (Range) kg	Gelding	Mare	Stallion
Warmblood	31	12 (7-28)	605 (464-710)	14	15	2
Icelandic horses	16	10 (4-23)	435.5 (316-510)	6	10	0
Quarterhorse	12	15 (3-27)	502.5 (430-595)	5	6	1
Thoroughbred	7	16 (12-31)	485 (344-507)	2	5	0
Pony	10	15 (9-30)	336.5 (167-554)	5	5	0
Draft horses	2	17.5 (16,19)	719 (703,735)	2	0	0

All treatments were well tolerated, and no complications occurred throughout the study. In five horses mouth opening to an ISD of 102 mm was impossible due to resistance and one horse had to be excluded due to extreme deviations in the results. Horses showed a median mandible length of 48 cm (IQR 4 cm). Regular dental treatment was recorded in 43 cases (61%), compared to 28 cases (39%) with no regular treatment. In six cases owners had no knowledge of the history of dental treatments. The dosage of sedatives administered in the study ranged between 0.008 and 0.025 mg/kg for detomidine hydrochloride and between 0.004 and 0.012 mg/kg for butorphanol tartrate with a ratio of 2:1.

**Figure 4.**
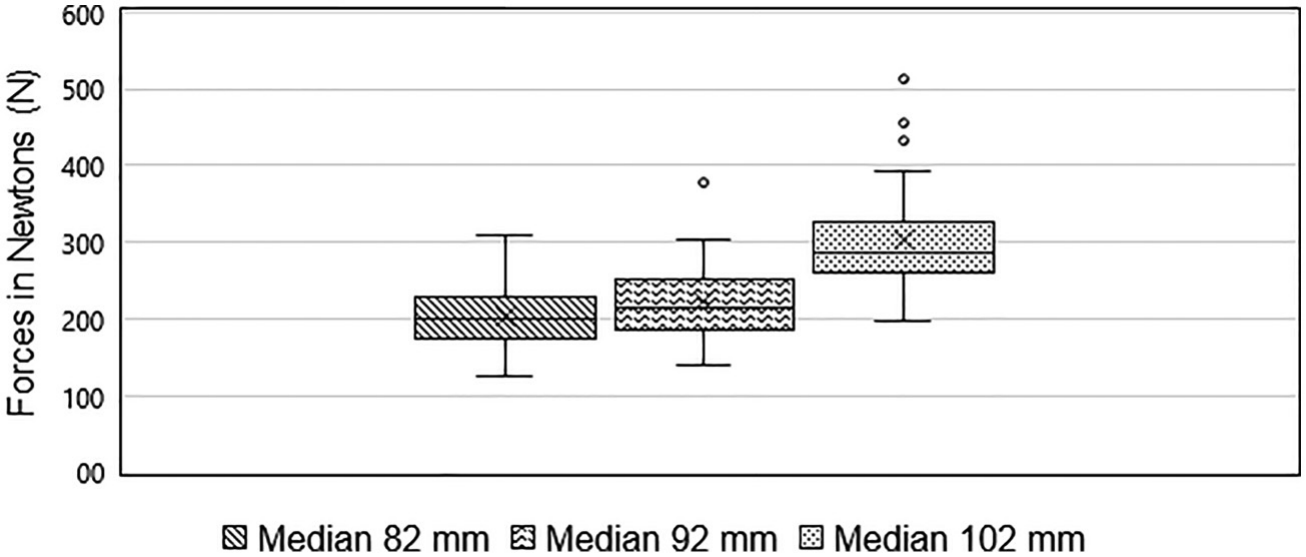
Boxplot of median forces during different incisor separation distances of the mouth speculum. The line in the boxplot represents the median, the boundaries represent the 25th and 75th percentiles. The “*x*” depicts the mean value.

Force measurements showed varying values with respect to the different ISD of the mouth speculum. A median of 198.88 N (IQR 56.44 N) for the ISD at 82 mm, 214.18 N (IQR 66.54 N) for 92 mm and 293.95 N (IRQ 112.80 N) for 102 mm was measured (Figure 4). At all three ISD, significant pairwise differences in the median forces (*z* = 110.11, *p* < .001) was demonstrated. The median forces at the lower ISD of 82 mm differ from those at 92 mm, in that they were lower (*z* = –2.833, *p *= .005). The force also differs between the lowest measured ISD (82 mm) to the highest measured ISD (102 mm). There was a lower force when the mouth was opened 82 mm (*z* = –10.167, *p* < .001). The median force at 92 mm is lower than at 102 mm (*z* = –7.333, *p *< .001). This data confirms the hypothesis that forces increased with increasing ISD of the horse's jaw.

**Figure 5.**
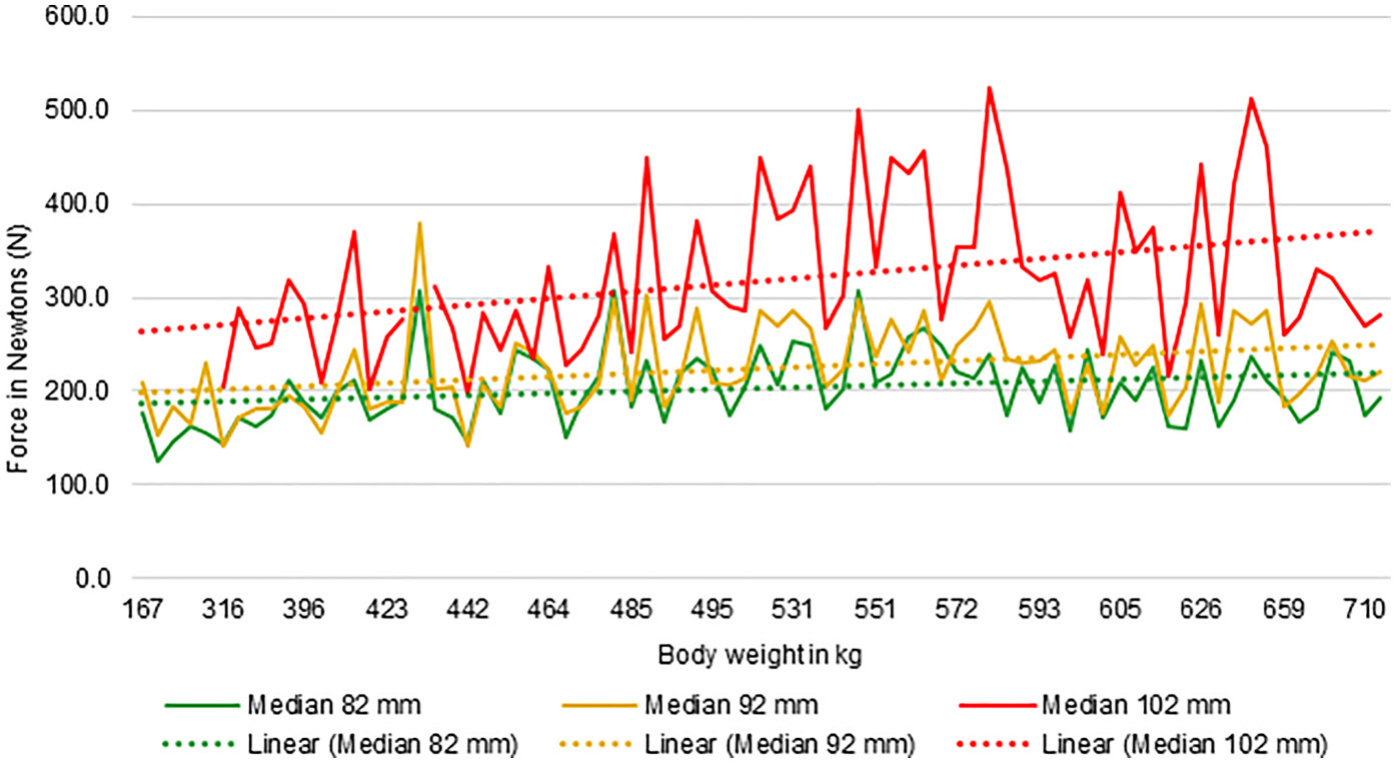
Graph showing the correlation between body weight in kg and forces in Newtons. The continuous lines show the median forces for 82 mm (green), 92 mm (orange) and 102 mm (red) incisor separation distance. The dotted lines are the corresponding trend lines.

The forces on the incisors increased with increasing body weight at an ISD of 82 mm (ρ=0.291, N = 77, *p *= .010), at an ISD of 92 mm (ρ=0.403, N = 77, *p *< .001) and at an ISD of 102 mm (ρ=0.383, N = 72, *p *= .001). The greatest correlation was noticed at an ISD of 92 mm (Figure 5).

With relation to mandible length, forces increased with increasing mandibular length. Mandible length demonstrated a positive correlation with the forces at an ISD of 92 mm (ρ=0.259, N = 77, *p *= .023) and 102 mm (ρ=0.334, N = 72, *p *= .004). There was no correlation between the length of the mandible and the forces exerted at the lowest ISD of 82 mm (ρ=0.166, N = 77, *p *= .149).

**Figure 6.**
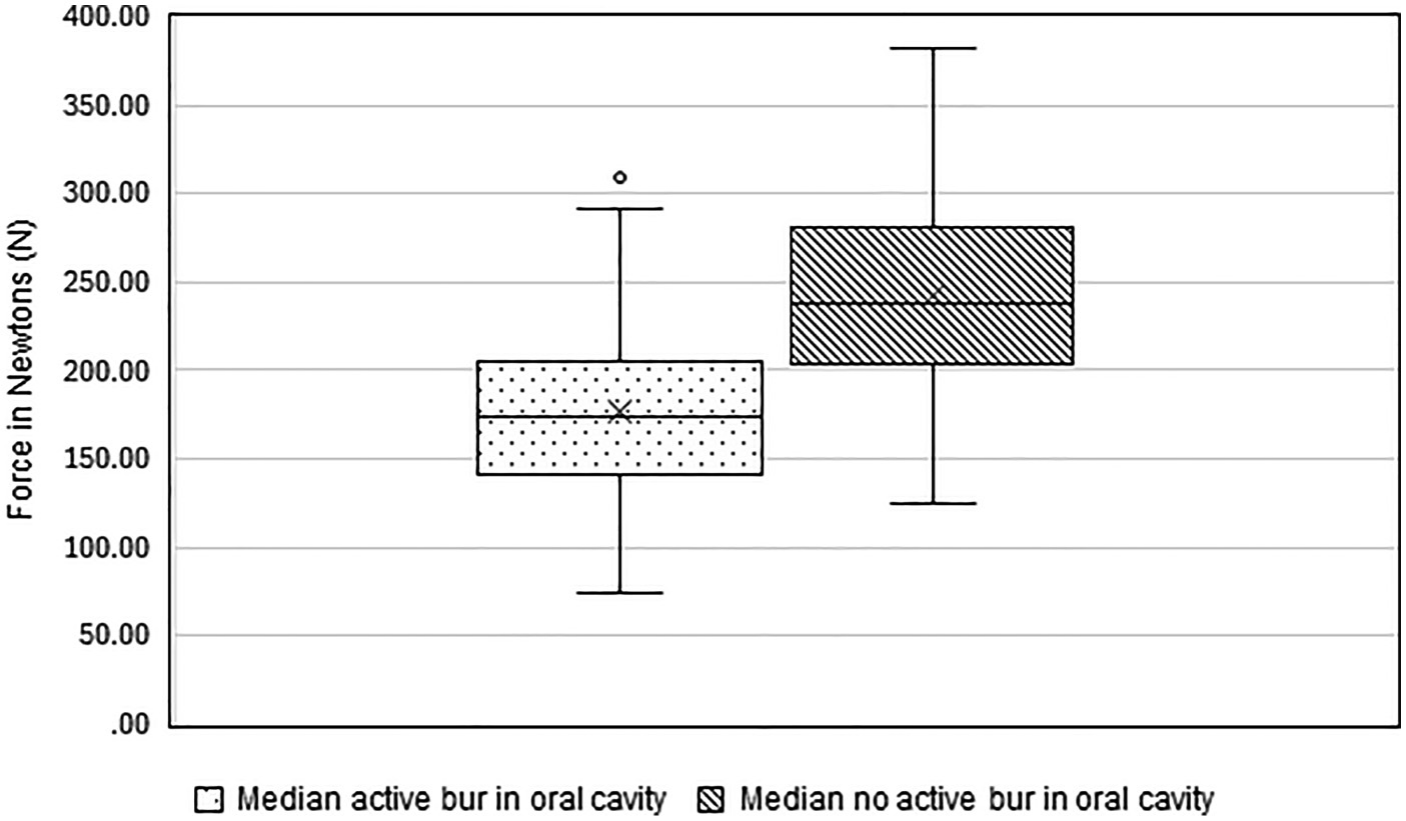
Boxplot of median forces with an active mechanical bur and no active mechanical bur during dental treatment. The line in the boxplot represents the median, the boundaries represent the 25th and 75th percentiles. The “*x*” depicts the mean value.

Measurements during dental treatment at an ISD of 92 mm showed a median force of 173.28 N (IQR 68.9 N) with an active mechanical bur within the horse's oral cavity and a median force of 237.81 N (IQR 78.75 N) with no active mechanical bur (Figure 6). Individual force maximums ranged from 304.69 N (31.07 kg) to 3783.60 N (385.82 kg) with a median maximal force of 1655.02 N (IQR 1223.35 N). There is a difference in measured forces between use of active versus nonactive mechanical burs (median) (*z* = –7.507, *p *< .001). The data does not support the hypothesis of higher occurring stresses while actively rasping the cheek teeth using a mechanical bur compared to stresses during examination. The forces during dental treatments were lower while actively rasping the cheek teeth with a bur than during examination.

A positive correlation was seen between mandible length and no use of an active mechanical bur (*p *< .001). However, there is no association between the mandible length and use of an active mechanical bur (*p *= .163).

To assess data regarding age and gender, as well as the influence of regular dental treatments independent of the size of the animals, the forces were converted to Newton/kg body weight. No correlation could be stated between age, gender or regular dental treatments and the resulting forces/kg body weight at the different ISD as well as forces/kg body weight during dental treatment.

## Discussion

This is the first study to describe forces on the incisors of horses during routine dental examination and odontoplasty of cheek teeth. Thus, no restrictions were made regarding horse breed and weight. A modified “Günther” mouth speculum was considered suitable due to the possibility of positioning the tension load cells in the direction of tension, almost eliminating possible measurement errors in the form of material friction losses.

The measurements were carried out in kilograms. They were then converted to newtons to enable comparison with the values published in the literature.^8-11^ This conversion has also been previously described in human medical publications.^13^

The large number of commercially available mouth specula allows a great variability in the possible ISD of the horse's jaw. Due to the practicability and experience of the authors, a decision was ultimately made to use the Hausmann mouth speculum as reference because the rachet system of the mentioned speculum allowed an exact determination of the ISD, and it is regarded as being widely used in veterinary practice. In the authors’ experience, oral examinations and dental treatments of most teeth are feasible at an ISD at ratchet 2 (82 mm). However, extension to ratchet 3 (102 mm) to treat distal molars was considered necessary at times. Opening the mouth speculum up to 102 mm generally results in considerable resistance. In five of the horses, it was impossible to open the speculum to 102 mm. To address this limitation but also allow adequate dental treatment of distal molars, a third measurement height at 92 mm was included.

The dosage of applied sedatives in this study showed a relatively wide range. The chosen dosage for each patient depended upon race-specific reactions to sedatives, individual animal-specific behavioral patterns, the environment and the knowledge of individual response due to previously performed dental treatments by the attending veterinarian (FS). This reflects daily veterinary practice and the acquired data is expected to mimic such regular situations.^14^

Measured forces are influenced by several factors. These include increasing resistance due to the stretching of the masticatory muscles (i.e., masseter, pterygoids, digastricus) during the opening of the mouth, as well as forces occurring due to the suspension of the head in the special halter during dental treatment. The presumably predominant cause is expected to be the actively exerted chewing force of the masticatory muscles.

Both human and equine medicine studies describe the masseter and pterygoid muscles as the main muscles for masticatory force.^15,16^ Based on this study, an increase in the ISD of 20 mm would result in a base force increase of 95.07 N. Nevertheless, the influence of changes in the ISD of the oral cavity on the maximum forces due to stretching of the muscles is currently still unknown.

Although the median values during dental treatments showed a significant difference in forces, the study results do not illustrate the difference in measured forces as obviously (Figure 3). The values obtained can be attributed to discomfort caused by the rasping of the teeth with the bur, resulting in active opening of the mouth to avoid the stimulus, leading to reduced force values. Higher values with no active mechanical bur could be interpreted as a reaction to the absence of the unpleasant stimulus followed by enhanced chewing activity. The median values during dental treatments measured in this study are lower than the previously described mean values for cheek teeth in the literature (248-554 N [peaks closing stroke], 875-1956 N [peaks power stroke]).^11^ However, considering the median of the immediate maximum forces of 1655.02 N on the incisors during dental treatment and comparing these with the mean force of 875 ± 278 N at the “power stroke” on the level of the third premolar teeth during mastication, these values clearly exceed the previously described values despite sedation.^10^ The reason for this may be that chewing movement is generated due to an extrinsic unpleasant stimulus.

Two previous studies have already described measurements of maximum forces on the incisors during mastication.^8,9^ While the former determined the maximum lifting force of the mandible (33-107 kg),^9^ the latter measured the maximal forces of every individual incisor (14-36 kg).^8^ A comparison of these data with the current study (31.07-385.82 kg) reveals a substantial difference between the forces already described during a “normal chewing act” and the measured values occurring during dental treatment.

The data showed no difference in the forces with regard to age and regularity of dental treatment. However, based on other studies, age-related changes of incisor shape and alignment result in higher stresses on the involved structures and could cause potentially pathological effects in an older horse population.^17,18^

A positive correlation between mandible length and the absence of active rasping was noted in this study. However, it should be kept in mind that the represented population differed greatly in terms of body size and weight. Nevertheless, similar results have already been described in dogs, where a correlation of bite forces to head shape and head length could already be confirmed.^19^ No correlation was found between gender and occurring forces. The low number of stallions (*n* = 3) in the study does not allow conclusions regarding the gender-typical stronger physiology of the masseter muscle.

The data also provides information about stress on mouth specula themselves and can be used in the veterinary industry to further develop data-based mouth specula in terms of material used and options for ISD of the horse's jaw. The authors recommend using scaling systems for additional information since lever systems and single or multiple screw systems allow easier and greater opening of the horse's jaw when compared to the ratchet systems.

This is the first study that determines forces on the incisors in horses under sedation before and during odontoplasty of cheek teeth. The load on incisors increases significantly with the ISD ranging from 198.88-293.95 N. The forces measured during oral examination exceeded those measured during active treatment such as using a mechanical bur and so revealed higher stresses during examination.

## Materials

- Maulgatter nach Günther Modell Joachim Brand, Pegasos 4D, Waldkirch, Germany
- Hottinger Brüel & Kjaer GmbH, Darmstadt, Germany
- Mouth speculum for Horses 605 4050, World Wide Equine (WWE), Inc., ID, USA
- Version 27, IBM Corp., Armonk, NY, USA.
